# Generation of Recombinant Authentic Live Attenuated Human Rotavirus Vaccine Strain RIX4414 (Rotarix^®^) from Cloned cDNAs Using Reverse Genetics

**DOI:** 10.3390/v16081198

**Published:** 2024-07-25

**Authors:** Saori Fukuda, Masanori Kugita, Kanako Kumamoto, Yuki Akari, Yuki Higashimoto, Shizuko Nagao, Takayuki Murata, Tetsushi Yoshikawa, Koki Taniguchi, Satoshi Komoto

**Affiliations:** 1 Department of Virology, Fujita Health University School of Medicine, Toyoake 470-1192, Aichi, Japan; saorif@fujita-hu.ac.jp (S.F.); m24d9001@oita-u.ac.jp (Y.A.); tmurata@fujita-hu.ac.jp (T.M.); kokitani@fujita-hu.ac.jp (K.T.); 2 Education and Research Facility of Animal Models for Human Diseases, Fujita Health University, Toyoake 470-1192, Aichi, Japan; m-kugi@fujita-hu.ac.jp (M.K.); kumamoto@fujita-hu.ac.jp (K.K.); shizun@fujita-hu.ac.jp (S.N.); 3 Division of One Health, Research Center for GLOBAL and LOCAL Infectious Diseases (RCGLID), Oita University, Yufu 879-5593, Oita, Japan; 4 Department of Clinical Microbiology, Fujita Health University School of Medical Sciences, Toyoake 470-1192, Aichi, Japan; yhigashi@fujita-hu.ac.jp; 5 Department of Pediatrics, Fujita Health University School of Medicine, Toyoake 470-1192, Aichi, Japan; tetsushi@fujita-hu.ac.jp; 6 Center for Infectious Disease Research, Research Promotion Headquarters, Fujita Health University, Toyoake 470-1192, Aichi, Japan

**Keywords:** human rotavirus, live attenuated rotavirus vaccine, Rotarix**^®^**, reverse genetics

## Abstract

The live attenuated human rotavirus vaccine strain RIX4414 (Rotarix**^®^**) is used worldwide to prevent severe rotavirus-induced diarrhea in infants. This strain was attenuated through the cell culture passaging of its predecessor, human strain 89-12, which resulted in multiple genomic mutations. However, the specific molecular reasons underlying its attenuation have remained elusive, primarily due to the absence of a suitable reverse genetics system enabling precise genetic manipulations. Therefore, we first completed the sequencing of its genome and then developed a reverse genetics system for the authentic RIX4414 virus. Our experimental results demonstrate that the rescued recombinant RIX4414 virus exhibits biological characteristics similar to those of the parental RIX4414 virus, both in vitro and in vivo. This novel reverse genetics system provides a powerful tool for investigating the molecular basis of RIX4414 attenuation and may facilitate the rational design of safer and more effective human rotavirus vaccines.

## 1. Introduction

Group A rotavirus (RVA), a member of *Sedoreoviridae*, is a primary cause of severe gastroenteritis in young children worldwide, accounting for approximately 128,500–215,000 deaths annually in children aged under 5 years of age [1,2]. The virion contains an 11-segment double-stranded RNA (dsRNA) genome encoding six structural proteins (VP1-VP4, VP6, and VP7) and six non-structural proteins (NSP1-NSP6) [3]. Each dsRNA segment possesses one or two protein-coding sequences flanked by untranslated regions (UTRs).

Currently, there are no specific antiviral treatments for RVA gastroenteritis, and prevention through vaccination remains the most effective approach. Live oral human RVA (HuRVA) vaccines have been developed through the serial passaging of clinical HuRVAs in cell cultures and were found safe in neonates and infants. Rotarix**^®^** (GlaxoSmithKline), one of the most widely used live attenuated vaccines globally, is licensed in >100 countries and universally recommended for all infants in many of these [4,5,6]. This vaccine is based on the RIX4414 (G1P[8]) strain, which was derived from the HuRVA strain 89-12 (G1P[8]), isolated from a child with diarrhea in the United States during the 1988–1989 rotavirus season. To develop RIX4414, strain 89-12 was passaged 33 times in primary African green monkey cells and further passaged in monkey Vero cells to acquire attenuating mutations in its genome [7]. Infants orally administered the serially passaged strain 89-12 (strain RIX4414) did not develop diarrhea, confirming its safety. However, the genetic basis for the attenuation of vaccine strain RIX4414 remains unknown. Elucidating these mechanisms at the molecular level will enhance our knowledge of RVA pathology and may contribute to the development of new vaccines.

The advances in reverse genetics have allowed RVA genomes to be artificially manipulated, significantly enhancing our ability to research these viruses. However, HuRVA strains typically do not grow as robustly in cell culture (~10^6^ plaque-forming units (PFU)/mL) as various animal RVA strains, such as simian SA11 virus (~10^8^ PFU/mL), making the development of reverse genetics systems more challenging for HuRVAs than for animal RVAs [8,9,10]. Despite these difficulties, reverse genetics systems have been developed for several HuRVA strains. Strain KU (G1P[8]) [11] was the first HuRVA to be successfully manipulated by reverse genetics to generate an infectious virus [8]. Subsequently, systems for a few other HuRVAs, including CDC-9 (G1P[8]) [9], HN126 (G2P[4]) [12], and Odelia (G4P[8]) [13], have been described. However, none of these HuRVA strains represent licensed, clinically validated attenuated vaccine strains. Given this, a reverse genetics platform based on authentic vaccine strain RIX4414 would be highly valuable, enabling targeted genetic manipulations to unravel the attenuation mechanisms of the live HuRVAs vaccines.

Very recently, the rescue of the recombinant RIX4414-like virus by reverse genetics was reported [14]. This RIX4414-like virus was mostly based on RIX4414 but incorporated segments of the 5′- and 3′-UTRs from the wild-type HuRVA strain Wa (G1P[8]) [15], a modification required because of incomplete sequence data for certain UTRs of strain RIX4414 in the GenBank/EMBL/DDBJ data libraries. Additionally, the RIX4414-like virus VP2 protein included residues shared with several wild-type HuRVAs (Wa (G1P[8]), KU (G1P[8]), and Odelia (G4P[8])) instead of those found in RIX4414 VP2, because the originally attempted rescue T7 plasmid carrying the VP2 gene of RIX4414 did not function in reverse genetics [14]. The UTR regions and VP2 core shell protein are involved in all stages of RVA replication and virion assembly [16,17,18,19,20,21]. Therefore, a more reliable and complete understanding of the molecular mechanisms of attenuation in vaccine strain RIX4414 necessitates a more authentic reverse genetics system.

In this study, we determined the complete sequences of all 11 dsRNA segments of RIX4414 using next-generation sequencing (NGS) and successfully developed a reverse genetics system that accurately represents the authentic RIX4414 virus. This system provides a valuable genetic platform for elucidating the attenuated pathogenic mechanisms of RIX4414.

## 2. Materials and Methods

### 2.1. Cells and Viruses

A baby hamster kidney cell line stably expressing the T7 RNA polymerase (BHK/T7-9) [22] was cultured in Dulbecco’s modified Eagle medium (DMEM; Nacalai, Kyoto, Japan) supplemented with 5% fetal calf serum (FCS; Gibco, Tokyo, Japan) (complete medium) in the presence of 600 ng/mL hygromycin (Invitrogen, Tokyo, Japan). Monkey kidney cell lines, MA104 and CV-1, were cultured in complete medium. HuRVA vaccine strain RIX4414 (G1-P[8]-I1-R1-C1-M1-A1-N1-T1-E1-H1) was obtained directly from a vial of Rotarix**^®^** (GlaxoSmithKline, Tokyo, Japan). Strain RIX4414 and recombinant simian RVA strain SA11-L2 (G3-P[2]-I2-R2-C5-M5-A5-N5-T5-E2-H5) (rSA11-L2) [23] were propagated as described previously [24]. Briefly, RIX4414 and rSA11-L2 viruses were pretreated with trypsin (type IX, from porcine pancreas; 10 μg/mL) (Sigma-Aldrich, Tokyo, Japan) and then propagated in MA104 cells in Eagle’s minimum essential medium (MEM; Nissui, Tokyo, Japan) without FCS (incomplete medium) but containing trypsin (1 μg/mL).

### 2.2. cDNA Library Construction, Illumina MiSeq Sequencing, and Sequence Analysis of RIX4414 Virus

Construction of a cDNA library and Illumina MiSeq sequencing for RIX4414 virus were conducted as described previously [25,26]. Viral genomic dsRNAs were directly extracted from a suspension of RIX4414 virus in a vial of Rotarix**^®^**, without any passaging in our laboratory, using a QIAamp Viral RNA Mini Kit (Qiagen, Tokyo, Japan). A 200-bp fragment cDNA library ligated with bar-coded adapters was prepared using an NEBNext Ultra RNA Library Prep Kit for Illumina v1.2 (New England Biolabs, Tokyo, Japan) according to the manufacturer’s instructions. The cDNA library was isolated using Agencourt AMPure XP magnetic beads (Beckman Coulter, Tokyo, Japan). After assessing the quality and quantity of the purified cDNA library, nucleotide sequencing was performed five times on an Illumina MiSeq sequencer (Illumina, Tokyo, Japan) using a MiSeq Reagent Kit v2 (Illumina) to generate 151 paired-end reads. Analysis of the MiSeq sequencing data was performed using a CLC Genomics Workbench v8.0.1 (CLC Bio, Tokyo, Japan). Contigs were assembled from the yielded sequence reads (trimmed) by de novo assembly. Using the assembled contigs as query sequences and the Basic Local Alignment Search Tool (BLAST, https://blast.ncbi.nlm.nih.gov/Blast.cgi) for searching the non-redundant nucleotide database of the National Center for Biotechnology Information (NCBI; https://www.ncbi.nlm.nih.gov/, accessed on 1 September 2018), it was determined which contigs represented the full-length nucleotide sequence for each segment of RIX4414 virus including the typical RVA-segment endings. The determined sequences have been deposited in GenBank/EMBL/DDBJ, and the accession numbers for the nucleotide sequences of the VP1-VP4, VP6, VP7, and NSP1-NSP5 genes of strain RIX4414 are LC822556-LC822566, respectively.

### 2.3. Construction of Rescue T7 Plasmids Carrying All 11 dsRNA Segments of RIX4414 Virus

To develop a reverse genetics system for the RIX4414 virus, we newly constructed 11 rescue T7 plasmids for transcription of each mRNA of the 11 dsRNA segments of RIX4414 virus. For this, full-length cDNA fragments matching the 11 dsRNA segments were biochemically synthesized by Eurofins Genomics (Tokyo, Japan) or GENEWIZ (Tokyo, Japan) based on the full-length genomic sequences of the RIX4414 virus determined in this study, and each was individually cloned into a pUC57-derived pUC57R vector that carries the antigenomic hepatitis delta virus (HDV) ribozyme and T7 RNA polymerase terminator sequences [23]. In each of the constructed T7 plasmids, a cDNA copy of a full-length dsRNA segment is flanked by the T7 RNA polymerase promoter and HDV ribozyme sequences [27], and followed by the T7 RNA polymerase terminator sequence. A rescue T7 plasmid containing a signature mutation to destroy the unique HindIII restriction enzyme site in the VP3 gene at position 1039 (by a synonymous mutation) was also constructed using artificial synthesis by GENEWIZ. The 12 rescue T7 plasmids containing the genome of strain RIX4414 were pT7/VP1RIX, pT7/VP2RIX, pT7/VP3RIX, pT7/VP3RIX-ΔHindIII, pT7/VP4RIX, pT7/VP6RIX, pT7/VP7RIX, pT7/NSP1RIX, pT7/NSP2RIX, pT7/NSP3RIX, pT7/NSP4RIX, and pT7/NSP5RIX. 

### 2.4. Reverse Genetics System

The protocol was basically as described previously [8,23]. For strain RIX4414, the above-described rescue T7 plasmids were employed. As a comparison, 11 rescue T7 plasmids encoding the genome of simian laboratory strain SA11-L2 [28] were also employed, namely, pT7/VP1SA11, pT7/VP2SA11, pT7/VP3SA11, pT7/VP4SA11-ΔPstI, pT7/VP6SA11, pT7/VP7SA11, pT7/NSP1SA11, pT7/NSP2SA11, pT7/NSP3SA11, pT7/NSP4SA11, and pT7/NSP5SA11 [23]. Briefly, the protocol was as follows. Monolayers of BHK/T7-9 cells in 6-well plates (Falcon, Bedford, MA, USA) were cotransfected with 11 T7 plasmids, representing the cloned cDNAs of 11 RVA dsRNA segments, in the following different quantities: pT7/VP1RIX (0.75 μg), pT7/VP1SA11 (0.75 μg), pT7/VP2RIX (0.75 μg), pT7/VP2SA11 (0.75 μg), pT7/VP3RIX-ΔHindIII (0.75 μg), pT7/VP3SA11 (0.75 μg), pT7/VP4RIX (0.75 μg), pT7/VP4SA11-ΔPstI (0.75 μg), pT7/VP6RIX (0.75 μg), pT7/VP6SA11 (0.75 μg), pT7/VP7RIX (0.75 μg), pT7/VP7SA11 (0.75 μg), pT7/NSP1RIX (0.75 μg), pT7/NSP1SA11 (0.75 μg), pT7/NSP2RIX (2.25 μg), pT7/NSP2SA11 (2.25 μg), pT7/NSP3RIX (0.75 μg), pT7/NSP3SA11 (0.75 μg), pT7/NSP4RIX (0.75 μg), pT7/NSP4SA11 (0.75 μg), pT7/NSP5RIX (2.25 μg), and/or pT7/NSP5SA11 (2.25 μg). To generate recombinant SA11-L2 x RIX4414 single segment-reassortant viruses, the rescue T7 plasmid encoding the segment of SA11-L2 virus, which was to be replaced, was exchanged with a rescue T7 plasmid representing the corresponding dsRNA segment of RIX4414 virus; this process was carried out for each of the 11 individual dsRNA segments. Following a 1-day incubation, the transfected BHK/T7-9 cells were washed with incomplete medium and then cocultured with overlaid CV-1 cells (5 × 10^4^ cells/mL) for 3 days in incomplete medium containing trypsin (0.3 or 0.9 μg/mL). After this, the cultures were subjected to two cycles of freezing and thawing and then treated with trypsin (10 μg/mL) for RVA activation, followed by inoculation onto MA104 cells in a roller-tube culture [29]; after one additional passage in such a culture and a 1-day incubation, recombinant RVAs were rescued and subsequently plaque purified in CV-1 cells, as previously described [30].

### 2.5. PAGE Analysis of Viral Genomic dsRNAs

Viral genomic dsRNAs were extracted from cell cultures using a QIAamp Viral RNA Mini Kit (Qiagen). The extracted viral genomic dsRNAs were subjected to polyacrylamide gel electrophoresis (PAGE) analysis: they were run in a 10% polyacrylamide gel for 16 h at 20 mA at room temperature, followed by silver staining [24] to visualize the genomic dsRNA migration profiles.

### 2.6. Multiple-Step Virus Growth

Monolayers of MA104 cells in 12-well plates (Thermo Fisher Scientific, Rochester, NY, USA) were infected in triplicate with trypsin-pretreated RVAs at an MOI of 0.01, washed twice with incomplete medium, and then incubated in incomplete medium containing trypsin (1 μg/mL) over various time periods. The infected cells were frozen and thawed twice before the measurement of viral titers by plaque assay.

### 2.7. Plaque Assay

The plaque assays were conducted as described previously [31]. Briefly, confluent monolayers of CV-1 cells in 6-well plates (Falcon) were infected with trypsin-pretreated RVAs, washed twice with incomplete medium, and then cultured with trypsin (1 μg/mL) in primary overlay medium (0.7% agarose). After 2 or 3 days, the cells were stained with secondary overlay medium containing 0.005% neutral red (Sigma-Aldrich) and 0.7% agarose. Plaque sizes were determined by measuring the mean diameters of 25 plaques in 2 independent assays.

### 2.8. Mouse Experiment

The mouse experiment was basically performed as previously described [32,33,34,35]. Briefly, pregnant BALB/cCrSlc mice (16 days of gestation) were purchased from Japan SLC Inc, Shizuoka, Japan. The mice were housed individually, and suckling mice were born on day 19.5 of gestation on average. To assess the rate and score of diarrhea, 5-day-old suckling mice were orally administered 50 μL of cell culture supernatant containing RIX4414, rRIX4414, or rSA11-L2 (1.0 × 10^5^ PFU/mouse), or cell culture medium without RVA (mock), and were then monitored daily over a period of 5 days for diarrhea following gentle abdominal palpation. Mock inoculations were performed using MEM without additives. Rating of diarrhea (diarrhea score) was performed based on the following scale: 0, no diarrhea (normal stool or no stool); 1, soft orange stool; 2, soft mucous stool; and 3, liquid stool [32,33]. In this study, the ‘diarrhea’ status was defined as diarrhea score: ≥1. To compare the pathological changes in intestinal tissues after RVA infection and confirm RVA infection in the small intestines, suckling mice at 2–4 days post-infection were subjected to analysis of histopathology and RVA antigen expression of the small intestines. The animal experiment protocol was approved by the Fujita Health University Animal Care and Use Committee (Approval No.: APU19037-MD2).

### 2.9. Histopathology and Immunochemistry of Small Intestines

Under anesthesia, the small intestines of RVA-inoculated suckling mice were harvested and fixed in 4% paraformaldehyde phosphate-buffered solution (FUJIFILM Wako Chemicals, Osaka, Japan) for 1 day, followed by preparation of paraffin blocks, hematoxylin and eosin (HE) staining, and immunohistochemistry. In immunohistochemistry, a major RVA antigen was detected using BOND RX stainer (Leica, Tokyo, Japan). Deparaffinized and rehydrated sections were used for detection of RVA VP6 protein by using a mouse monoclonal antibody recognizing VP6 protein (YO-156 antibody [36]). HE-stained sections and immunostained sections were used for observation of RVA-induced lesions and VP6 protein expression, respectively, using a BX51 optical microscope (Olympus, Tokyo, Japan).

### 2.10. Statistics

Virus titers were evaluated by means of a two-way ANOVA with Sidak’s post-test. Statistical analyses were completed using GraphPad Prism 7 (GraphPad Software 7, Boston, MA). *p* values of < 0.05 were considered statistically significant.

## 3. Results

### 3.1. Sequence Determination of the Full RIX4414 Genome

Sequence information for the 5′- and/or 3′-UTRs of multiple segments is absent in the available sequence reports for the authentic RIX4414 virus (“Rotarix-A41CB052A”) in the GenBank/EMBL/DDBJ data libraries (JN849113, JN849114, and KX954616-KX954624), reflecting a focus on the gene coding sequences by Zeller et al. [37,38]. The reported lengths of the sequences for VP1-VP4, VP6, VP7, and NSP1-NSP5 of Rotarix-A41CB052A virus are 3267, 2673, 2508, 2359, 1194, 978, 1461, 954, 933, 528, and 594 nucleotides, respectively. In this study, we determined the complete nucleotide sequences of all 11 dsRNA segments of vaccine RIX4414 virus in a vial of Rotarix**^®^** by performing deep sequencing with Illumina MiSeq. This approach allowed the determination of the complete nucleotide sequences of all 11 gene segments of the authentic RIX4414 virus. The lengths in nucleotides of the VP1-VP4, VP6, VP7, and NSP1-NSP5 dsRNA segments of the RIX4414 were found to be 3302, 2717, 2591, 2359, 1356, 1062, 1566, 1059, 1074, 750, and 664, respectively. All the determined genetic sequences of the RIX4414 were identical to those of the Rotarix-A41CB052A, the exception being the VP4 gene that showed a one nucleotide difference (the residues are R and G at nucleotide position 1112 for Rotarix-A41CB052A and RIX4414, respectively). The genotype constellation of RIX4414 was identified as G1-P[8]-I1-R1-C1-M1-A1-N1-T1-E1-H1.

### 3.2. Construction of 11 Rescue T7 Plasmids for Live Attenuated Vaccine RIX4414 Virus

**Figure 1:**
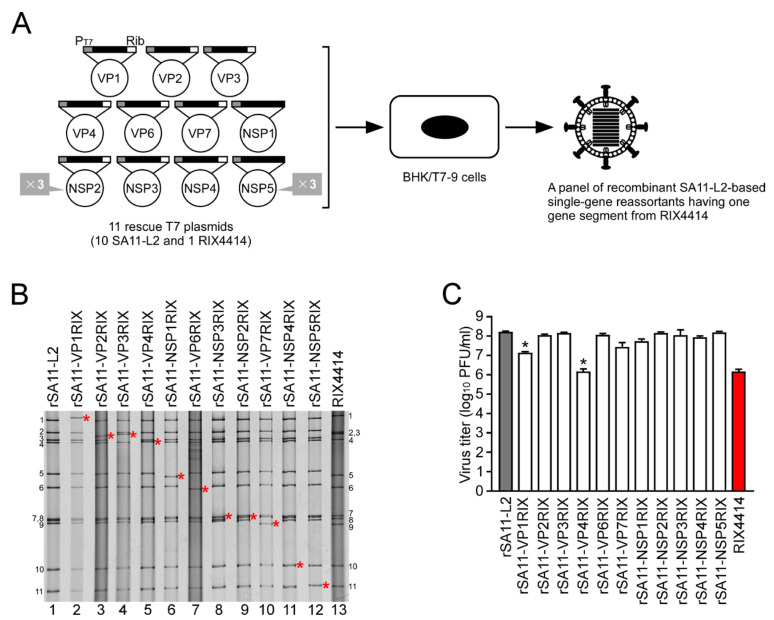
Generation of a panel of recombinant SA11-L2-based single-segment reassortants having one gene segment from RIX4414. (**A**) Schematic presentation of an 11-plasmid reverse genetics system to generate SA11-L2-based single-segment reassortants. The 11 rescue T7 plasmids include the full-length segment of cDNA of each dsRNA segment of RVA, flanked by the T7 RNA polymerase promoter (PT7) and the HDV ribozyme (Rib). To generate SA11-L2-based single-segment reassortants having one gene segment from RIX4414, BHK/T7-9 cells were cotransfected with the 11 rescue T7 plasmids (10 for SA11-L2 plus one for RIX4414) with 3-fold increased amounts of the two plasmids carrying the NSP2 and NSP5 genes. A panel of recombinant SA11-L2-based single-segment reassortants having one segment from RIX4414 were rescued from the cultures of the transfected BHK/T7-9 cells. (**B**) PAGE analysis of recombinant SA11-L2-based single-segment reassortants with each of the 11 segments from RIX4414. Lanes 1 and 13, dsRNAs from rSA11-L2 (lane 1) and RIX4414 (lane 13); lanes 2–12, dsRNAs from rescued rSA11-VP1RIX (lane 2), rSA11-VP2RIX (lane 3), rSA11-VP3RIX (lane 4), rSA11-VP4RIX (lane 5), rSA11-NSP1RIX (lane 6), rSA11-VP6RIX (lane 7), rSA11-NSP3RIX (lane 8), rSA11-NSP2RIX (lane 9), rSA11-VP7RIX (lane 10), rSA11-NSP4RIX (lane 11), and rSA11-NSP5RIX (lane 12). Red asterisks indicate the positions of the cDNA-derived RIX4414 segments. The numbers on the left and right indicate the orders of the genomic dsRNA segments of rSA11-L2 and RIX4414, respectively. (**C**) Infectivity of recombinant SA11-L2-based single-segment reassortants with each of the 11 segments from RIX4414. MA104 cells were infected with RVAs at an MOI of 0.01 and then incubated for 36 h. The viral titers in the cultures were determined by plaque assay. The data shown are the mean viral titers and standard deviations (SDs) for three independent cell cultures. Asterisks indicate significant differences between rSA11-L2 and recombinant single-segment reassortants; *, *p* < 0.05 (as calculated by two-way ANOVA with Sidak’s post-test).

Our strategy for constructing 11 rescue T7 plasmids for the RIX4414 virus was essentially based on the approach used for the wild-type HuRVA strain KU, which established the first HuRVA reverse genetics system [8]. To generate recombinant authentic RIX4414, 11 T7 rescue plasmids were created, each designed to express mRNA corresponding to one of the full-length dsRNA segments as determined by our sequencing analysis. These segment sequences were synthesized biochemically and cloned into individual T7-driven plasmids, flanked by the T7 RNA polymerase promoter and HDV ribozyme sequences (Figure 1A).

### 3.3. Generation of a Panel of Recombinant SA11-L2-Based Single-Segment Reassortant Viruses Carrying One RIX4414-Derived Segment

To individually access the functionality of each of the newly constructed 11 rescue T7 plasmids encoding the RIX4414 virus genome, we generated a panel of recombinant SA11-L2 x RIX4414 single-segment reassortant viruses. These viruses utilized a simian SA11-L2 genetic backbone, consisting of 10 T7 plasmids that each encoded a different SA11-L2 dsRNA-segment, and one T7 plasmid encoding the RIX4414 version of the missing dsRNA-segment. These 11 T7 plasmids were co-transfected into BHK/T7-9 cells using the 11 plasmid-based reverse genetics system (Figure 1A) [8,23]. For each of the 11 individual RIX4414 T7 plasmids, this method successfully produced recombinant SA11-L2-based single-segment reassortant viruses, thereby demonstrating the functionality of all the plasmids in reverse genetics.

In PAGE analysis, the viral genomic dsRNAs extracted from the 11 rescued recombinant single-segment reassortants displayed migration patterns where each respective RIX4414 segment was aligned with the corresponding segment in the RIX4414 virus (Figure 1B). Furthermore, the nucleotide sequence analysis of the extracted viral genomic dsRNAs validated the authenticity of each manipulated genomic segment. Thus, these results collectively confirm the functionality in reverse genetics of all 11 rescue T7 plasmids for RIX4414 that we constructed.

To estimate the growth potential of the rescued 11 recombinant SA11-L2-based single-segment reassortants, viral titers of rSA11-L2, the 11 recombinant SA11-L2 x RIX4414 single-segment reassortants, and RIX4414 were determined at 36 h after infection of MA104 cells at an MOI of 0.01 PFU/cell (Figure 1C). Nine single-segment reassortants, rSA11-VP2RIX, rSA11-VP3RIX, rSA11-VP6RIX, rSA11-VP7RIX, rSA11-NSP1RIX, rSA11-NSP2RIX, rSA11-NSP3RIX, rSA11-NSP4RIX, and rSA11-NSP5RIX, exhibited virus growth similar to rSA11-L2. On the other hand, two reassortants, rSA11-VP1RIX and rSA11-VP4RIX, exhibited impaired growth, with titers that were ~10-fold and ~100-fold lower, respectively. This is consistent with previous evidence that the HuRVA-derived spike VP4 proteins are associated with reduced viral growth in cell culture [8,9,12,13,24,28,39,40]. The involvement of HuRVA-derived RNA-dependent RNA polymerase VP1 proteins as a determinant of viral growth in cell culture was reported for HuRVA strains KU (G1P[8]), CDC-9 (G1P[8]), and Odelia (G4P[8]), but not for HuRVA strain HN126 (G2P[4]) [9,12,13,39], suggesting a possible sub-optimal protein interaction between the exchanged HuRVA-derived VP1 protein and the other existing structural and/or non-structural proteins of simian SA11-L2 virus in a strain-specific fashion.

### 3.4. Generation of Recombinant Authentic Live Attenuated Vaccine Strain RIX4414 (rRIX4414) from Cloned cDNAs

We proceeded using the 11 rescue T7 plasmids that encode the RIX4414 genome to generate a replicative, authentic RIX4414 virus. Notably, for marking purposes, instead of pT7/VP3RIX plasmid, we used a pT7/VP3RIX-ΔHindIII plasmid, in which a unique HindIII site in the VP3 gene was eliminated via a silent G-to-A mutation as described in the Materials and Methods section. The 11 plasmids were co-transfected into BHK/T7-9 cells using a modified 11-plasmid reverse genetics system for HuRVAs [8]. While no striking cytopathic effect (CPE) was observed in the first passage of the virus in MA104 cells using lysates of the cocultures of transfected BHK/T7-9 cells and overlaid CV-1 cells, a typical RVA CPE appeared in the MA104 cells during the second virus passage. This indicated the successful generation of recombinant authentic RIX4414 virus, named rRIX4414, entirely from cloned cDNAs.

**Figure 2:**
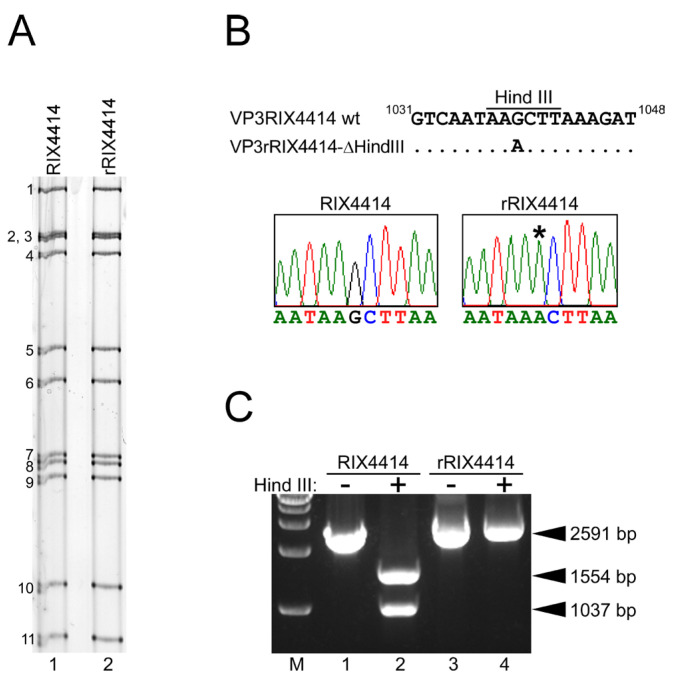
Generation of recombinant authentic rRIX4414 virus entirely from cloned cDNAs. (**A**) PAGE of viral genomic dsRNAs extracted from the parental RIX4414 and rescued rRIX4414. Lane 1, dsRNAs from the parental RIX4414; lane 2, dsRNAs from rescued rRIX4414. The numbers on the left indicate the order of the genomic dsRNA segments of RIX4414. (**B**) Rescued rRIX4414 contains a signature mutation (synonymous mutation) in its VP3 gene, because a nucleotide substitution (G-to-A at nucleotide position 1039) was introduced to abolish a unique HindIII site. The VP3 genes of RIX4414 and rRIX4414 were amplified by RT-PCR using specific primers, and sequencing electrograms show that indeed rRIX4414 possesses a G-to-A mutation at nucleotide position 1039. An asterisk indicates the G-to-A mutation introduced into the VP3 gene of rRIX4414. (**C**) Confirmation of the expected susceptibility to HindIII digestion. The 2591-bp VP3 gene RT-PCR products obtained for RIX4414 (lanes 1 and 2) and rRIX4414 (lanes 3 and 4) were treated with HindIII (+) or not (−), and separated in a 1% agarose gel. M, 1-kb DNA ladder marker.

To validate the genetic integrity of rRIX4414, we conducted PAGE, sequence, and restriction analyses. PAGE analysis of the viral genomic dsRNAs extracted from the rescued virus showed that rRIX4414 exhibited an RNA migration pattern indistinguishable from that of the parental RIX4414 virus (Figure 2A). To confirm that the rescued virus was generated from the cloned cDNAs, we confirmed the absence of a unique HindIII site, a marker genetic mutation introduced into the VP3 gene of rRIX4414 (Figure 2B, upper panel). The sequence analysis demonstrated that the VP3 gene from rRIX4414 had the introduced mutation at nucleotide position 1039, whereas the VP3 gene amplified from the parental RIX4414 virus did not (Figure 2B, lower panel). Furthermore, RT-PCR products derived from the VP3 gene of rRIX4414 were resistant to HindIII digestion, unlike those from the parental RIX4414 strain (Figure 2C). Whole-genomic sequencing using Illumina MiSeq also confirmed the presence of the expected G-to-A mutation in the VP3 gene and verified the absence of additional mutations across all 11 segments of the rescued rRIX4414 virus. These results confirm that the recombinant authentic RIX4414 virus was successfully generated via reverse genetics.

### 3.5. Characterization of rRIX4414 Virus in Cultured Cells

**Figure 3:**
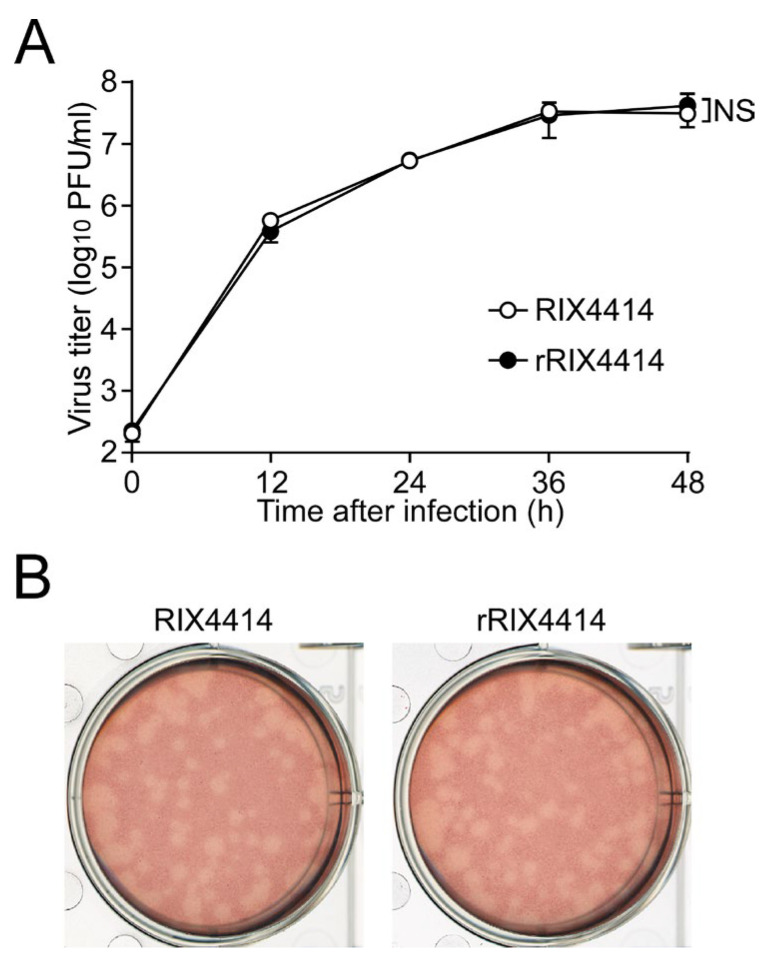
Growth properties of rRIX4414 virus in cultured cells. (**A**) Multiple-step growth curves for RIX4414 and rRIX4414. MA104 cells were infected with RIX4414 or rRIX4414 at an MOI of 0.01 and then incubated for various times (0, 12, 24, 36, and 48 h). The viral titers in the cultures were determined by plaque assay. The data shown are the mean viral titers and SDs from three independent cell cultures. NS, *p* > 0.05 (as calculated by two-way ANOVA with Sidak’s post-test). (**B**) Plaque formation by RIX4414 and rRIX4414. RIX4414 or rRIX4414 was directly plated onto CV-1 cells to form plaques. The experiment was repeated three times with similar results, and representative results are shown.

To evaluate whether the rescued rRIX4414 virus possesses the replication characteristics of the parental RIX4414 virus, multiple-step growth curves for RIX4414 and rRIX4414 were determined after infection of MA104 cells at an MOI of 0.01 PFU/cell. The growth curves showed that the replication of rRIX4414 was virtually identical to that of the parental RIX4414 (Figure 3A). We also compared the plaque sizes in CV-1 cells for these viruses by measuring the mean diameters of 25 plaques each in two independent assay repeats (examples in Figure 3B); this revealed that the rRIX4414 virus produced plaques of virtually the same size (diameter, 2.01 ± 0.46 mm) as those produced by the parental RIX4414 (diameter, 2.05 ± 0.42 mm). These results demonstrate that the replication characteristics of rRIX4414 in cultured cells are indistinguishable from those of the parental RIX4414.

### 3.6. Characterization of rRIX4414 Virus in Suckling Mice

**Figure 4:**
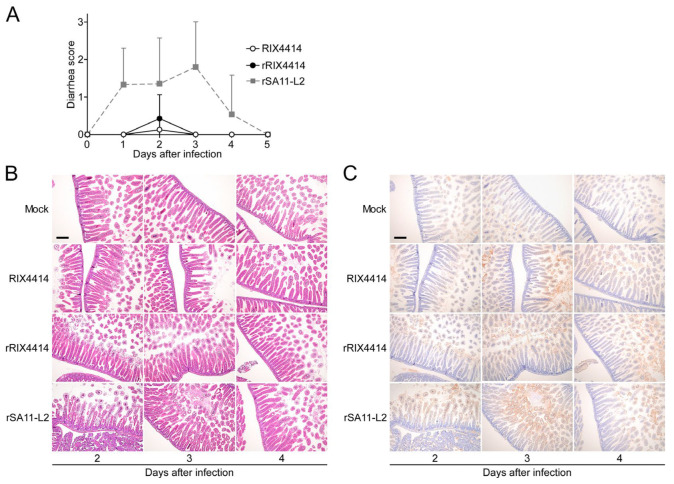
Biological properties of rRIX4414 virus in suckling mice. Five-day-old suckling mice were orally administered 50 μL of cell culture supernatants containing RIX4414, rRIX4414, rSA11-L2, or a mock infection. (**A**) Induction of diarrhea after infection with RIX4414, rRIX4414, or rSA11-L2. Diarrhea scores for individual mice were monitored daily. The data presented are the mean diarrhea scores and SDs for 9–28 pups. (**B**) Cytological changes in the small intestines. Small intestines were removed daily from day 2 to 4 after infection. Paraffin-embedded sections were stained with HE reagent. Two pups from each RVA-infected group were sacrificed for HE staining with similar results, and representative results are shown. From the mock-infection group, only one pup was sacrificed for HE staining. The bar represents 200 μm. (**C**) Histological analysis of rotaviral antigen expression in the small intestines. Small intestines of the infected mice were prepared as described in (**B**). Sections were stained by immunohistochemistry using a monoclonal antibody recognizing RVA VP6 antigen. VP6 protein expression in villus enterocytes was detected. The bar represents 200 μm.

To estimate whether the rescued rRIX4414 virus also possesses the biological characteristics of the parental RIX4414 virus in vivo, 5-day-old suckling mice were orally infected with RIX4414 or rRIX4414. To the best of our knowledge, there was no prior information about the pathogenicity of the vaccine strain RIX4414 in animal models including suckling mice, and therefore rSA11-L2 was included as a positive control known to induce diarrhea in suckling mice [32,33,35]. The RVA-inoculated mice were observed daily to check the possible onset of diarrhea according to previously established criteria [32,33]. Diarrhea was observed in the mice infected with RIX4414 and rRIX4414 solely on day 2 after infection, while those infected with rSA11-L2 experienced diarrhea on days 1–4 after infection (Table 1). In the mice infected with rSA11-L2, the average diarrhea score during days 1–5 was +1.26, whereas the average diarrhea scores in the same period were only +0.03 and +0.11 in the mice infected with RIX4414 and rRIX4414, respectively (Figure 4A). This indicates a reduced pathogenicity of RIX4414 and rRIX4414 relative to SA11-L2 in suckling mice.

We examined the pathological changes in the small intestine of infected mice on days 2–4 after infection by using HE staining of thin section preparations. Unlike the mock-infected mice, which showed no histological changes, significant epithelial vacuolization and villus shortening were observed in the small intestines of RVA-infected mice (Figure 4B). Notably, epithelial vacuolar degradation, a hallmark of RVA infection, was only observed on day 2 in the mice infected with RIX4414 or rRIX4414, but persisted throughout days 2 to 4 after infection in the rSA11-L2-infected mice.

To confirm the RVA infection in the small intestine, immunohistochemistry was performed using a mouse monoclonal antibody recognizing the VP6 protein, a major RVA antigen. On days 2–4 after RVA administration, the VP6 protein was detected in epithelial cells in the upper part of the villi of RVA-infected mice and not in mock-infected mice (Figure 4C). A visual estimation of the numbers of small intestine villus epithelial cells that expressed VP6 could not determine clear differences between rRIX4414-, RIX4414-, or rSA11-L2-infected mice.

The important conclusion from the combined results is that the biological characteristics of rRIX4414 in suckling mice are comparable to those of the parental RIX4414.

## 4. Discussion and Conclusions

We successfully established an authentic reverse genetics system for the HuRVA vaccine strain RIX4414. The recombinant strain, rRIX4414, exhibited properties both in vitro and in vivo that were indistinguishable from those of the original RIX4414 strain.

One of the limitations of this study is the comparison of the pathogenicity of rRIX4414 and RIX4414 with rSA11-L2 in suckling mice. Both HuRVA viruses, rRIX4414 and RIX4414, belong to the G1P[8] genotype, while the simian rSA11-L2 virus belongs to the G3P[2] genotype. This distinction is relevant for RVA infection because the P[8]-VP4 spike proteins recognize fucosylated histo-blood group antigens such as H1 antigen, while the P[2]-VP4 spike proteins recognize sialic acid present in gangliosides [41,42,43,44,45,46]. Therefore, to confirm the attenuated phenotype of rRIX4414, it would have been preferable to compare the vaccine strains with a human strain of the same P[8] genotype (e.g., the virulent Wa strain (G1P[8])). In any case, it was observed that the biological characteristics of rRIX4414 in suckling mice are comparable to those of the parental RIX4414 and that they were very mild compared to a more virulent RVA.

One future application of the recombinant rRIX4414 system is the further improvement of HuRVA vaccines. Currently, four live attenuated HuRVA vaccines are licensed and WHO-prequalified: Rotarix^®^ (G1P[8]), RotaTeq^®^ (five reassortant viruses; G1, G2, G3, G4, and P[8]), ROTAVAC^®^ (G9P[11]), and ROTASIIL^®^ (five reassortant viruses; G1, G2, G3, G4, and G5). Although these vaccines generally provide cross-protection against heterotypic HuRVA strains, outbreaks caused by strains heterotypic to the vaccines still occasionally occur [47,48,49,50,51], and wider vaccine immunogenicity would be advantageous. Additionally, despite several attempts, the attenuating mutations in the genome of RIX4414 have not yet been identified [52,53], and knowing them could benefit the development of other RVA vaccines. Furthermore, while the Rotarix^®^ vaccine is safe and well-tolerated [54], its vaccine strain RIX4414 is still capable of reversion to increased virulence due to mutations [55,56,57,58]. Hence, the identification and modification of these risk factors by using our established RIX4414 reverse genetics system may yield a safer vaccine strain. In short, we envision that the rRIX4414 reverse genetics platform will not only help uncover why RIX4414 is attenuated but will also serve as a base for rationally enhancing vaccine safety and immunogenicity.

Another long-term goal of the rRIX4414 platform is to develop an RVA-based enteric delivery vector. Since the advent of reverse genetics systems for RVA, it has been speculated that recombinant RVA could be used as a vector to deliver foreign genes to intestinal cells. Given the safety profile of Rotarix^®^, which is a vaccine licensed by the WHO, the rRIX4414 system is ideally positioned for exploring such possibilities.

## Figures and Tables

**Table 1:** Pathogenicity of RIX4414, rRIX4414, and rSA11-L2 viruses in 5-day-old suckling mice.

Days after Infection	RIX4414	rRIX4414	rSA11-L2	Mock
0	0% (0/23) *	0% (0/28)	0% (0/21)	0% (0/11)
1	0% (0/23)	0% (0/28)	66.7% (12/18)	0% (0/11)
2	13.0% (3/23)	35.7% (10/28)	58.8% (10/17)	0% (0/11)
3	0% (0/21)	0% (0/26)	73.3% (11/15)	0% (0/10)
4	0% (0/19)	0% (0/24)	23.1% (3/13)	0% (0/9)
5	0% (0/17)	0% (0/21)	0% (0/11)	0% (0/9)

* Rate of diarrhea (No. of diarrhea/inoculated).

## Data Availability

The nucleotide sequence data obtained in this study have been deposited in the DDBJ and EMBL/GenBank data libraries. The accession numbers for the nucleotide sequences of the VP1-VP4, VP6, VP7, and NSP1-NSP5 genes of strain RIX4414 are LC822556-LC822566, respectively.
